# A novel bridge wind-induced vibration response prediction algorithm based on temporal convolution network

**DOI:** 10.1371/journal.pone.0336973

**Published:** 2026-02-23

**Authors:** Youlai Qu, Xiangrong Bai, Tianhao Zhu, Shixu Zuo

**Affiliations:** 1 Power China Broadbridge Group Co. Ltd, Urumqi, Xinjiang, China; 2 School of Civil Engineering and Transportation, Hebei University of Technology, Tianjin, China; 3 School of Artificial Intelligence and Data Science, Hebei University of Technology, Tianjin, China; University of Zanjan, IRAN, ISLAMIC REPUBLIC OF

## Abstract

The stiffness of the high pier, large span rigid bridge in the operation period increases its ability to resist wind-induced vibration. However, the structural properties of high piers and long cantilevers make it susceptible to wind-induced vibration during construction in solid wind areas, which brings safety risks. The wind vibration response has strong nonlinear and random fluctuation characteristics, which brings significant challenges to the accurate prediction during the construction stage of bridges. A novel prediction algorithm for bridge wind-vibration response based on a temporal convolutional network (TCN) is proposed in this paper. It employs causal convolution to mine the mapping relationship of wind-induced vibration response acceleration data, utilizes dilation convolution to capture the multi-scale features of wind vibration response, and mitigates the gradient vanishing problem by residual connections between network layers. The proposed wind-induced vibration response prediction model based on TCN for bridges is compared in detail with advanced algorithms such as recurrent neural network (RNN), long-short-term memory network (LSTM), and gated unit network (GRU). The results demonstrate that the proposed algorithms have excellent prediction accuracy and generalization ability for wind vibration acceleration in different directions, such as torsion, vertical, transverse bridge, and along the bridge.

## 1. Introduction

With the continuous development of the economy in recent years, the construction scale of China’s transportation infrastructure continues to grow. As an essential transportation infrastructure, bridges are widely distributed in the western region. The airflow is blocked due to the topographic effect of the mountain passes in the canyon area, resulting in an increase in wind speed of 10% to 20%. The structural integrity of a high pier, large span continuous rigid bridge enhances its spanning capacity, and the construction is relatively simple, so it is widely employed in the construction of bridges in the strong wind region.

Compared with the construction stage, the overall stiffness of the high pier large span rigid-frame bridge in the operation period is larger and more resistant to wind-induced vibration. The characteristics of high piers and long cantilevers are susceptible to wind vibration, which can seriously slow down the construction progress and even lead to the overall destruction of the structure [1]. Consequently, accurate prediction of wind vibration response of large-span bridges contributes to the timely adoption of reasonable countermeasures. It has significant significance for guaranteeing safety during bridge construction and improving the efficiency of bridge construction.

The bridge-vibration response prediction algorithms have attracted the attention of many scholars and emerged as the current research hotspot of bridge preventive maintenance. It primarily contains two categories of prediction methods, which are based on statistical theory and data-driven. The statistical-based prediction approach has a more rigorous mathematical theory foundation and was widely employed in the field of bridge wind vibration prediction in the early stage [2–4]. Su et al. [5] identified comprehensive transfer functions based on segmental model vibrometry tests to achieve bridge vibration response prediction. Cui et al. [6] evaluated the probability of occurrence of vortex-excited vibration based on Fokker-Planck-Kolmogorov equations. Wang et al. [7] simulated the aeroelastic vibration of a bridge cross-section with computational fluid dynamics (CFD). Zhao et al. [8] and Zhang et al. [9] construct a nonlinear reduced-order model based on LSTM to improve the efficiency and accuracy of bridge aerodynamic prediction for flutter analysis under complex wind speed and nonlinear effects.A multi-scale approach derived the frequency response equations and the critical equations of vortex-excited vibration. Xu et al. [10] extended the vibration measurements at finite positions to the unmeasured region based on the method of modal superposition (MSM) and combination of beam functions (CMOBF) to predict the vibration response of cantilever plates. Li et al. [11] employed the Modal Superposition Method (MSM) and Combined Beam Function Method (CMOBF) to extrapolate vibration data from localized regions to unmeasured areas for predicting the response of cantilever plates. However, the modeling parameters such as bridge aerodynamic derivative function and chattering derivative are hardly to be accurately quantified in practical engineering due to the diversity of bridge cross-sections and the complexity of airflow [12–14]. Besides, the complicated mathematical formulas and weak generalization ability of the prediction model make it difficult to meet the performance requirements of wind vibration response prediction algorithms for cantilever bridges with variable cross-sections [15].

With the rapid development of machine learning theory, data-driven prediction methods have gradually emerged as the mainstream trend in bridge vibration prediction research. The core concept is to predict wind vibration response by mining the implicit features in the massive historical data of bridge vibration monitoring, which provides a novel idea for the prediction of wind vibration of bridges based on the structural dynamical response analysis model [16–18]. Ge et al. [19] predicted the probability of occurrence of vortex-excited vibration based on a decision tree that takes into account the effects of the structural damping ratio, wind direction, and wind speed. Tinmitondé et al. [20] proposed a probabilistic machine learning hierarchical Bayesian modeling method (PML-HBM). Zhang [21] employed a random forest(RF) algorithm based on Bayesian optimization to predict the vibration response of bridges induced by typhoons. The algorithm exhibited excellent performance in terms of accuracy and computational cost.. Xu et al. [22] investigated the effect of turbulence skewness and kurtosis based on ANN and virtual process method (VPM) on the long-term extreme value distribution of bridges. Ye et al. [23] proposed a bridge vibration prediction algorithm based on RF and Gaussian Mixture Model (GMM) and constructed vertical and lateral vibration early warning systems. Castello et al. [24] proposed a multi-layer perception prediction model based on support vector regression with multi-scale measurement data. The machine learning algorithm has excellent generalization ability and high prediction accuracy for different bridge excitations [25–27]. Li et al. [28] presented a Bayesian deep learning method for stochastic vibration analysis of bridges under coupled vehicle loads. The proposed approach is robust to data pollution, vehicle speed variation, and small dataset. Zhang et al. [29] proposed a novel graph isomorphic network (GIN) endowed with the self-evolutionary capability to predict new engineering scenarios based on existing data. Xiang et al. [30] proposed a graph-based framework to transfer features between components, which resembles real-world force transfer. The model achieves high prediction accuracy and robust generalization of the response to complex structures. Zhang et al. [31] further enhanced the GIN model by representing structures as graphs, enabling good generalization for unknown structural forms. Zhang et al. [32] proposed a hybrid convolutional neural network-long short-term memory(CNN-LSTM) model to improve the precision of fiber grating monitoring for high-speed railroads. A CNN-BiLSTM-attention hybrid neural network was proposed in ref [33], which can better capture the temporal information of the strain response. Zhang et al. [23] combined the CNN-LSTM model with ANFIS to leverage the strengths of both models. Xiang et al. [34] developed a deep learning framework for predicting stochastic seismic response using LSTM. The comparative analysis shown that the LSTM has significant prediction accuracy and efficiency in the seismic response.

The prediction accuracy of traditional machine learning methods, such as decision trees, Bayesian optimization random forests, and VPM, often fails to meet the actual performance requirements due to the solid nonlinear and non-stationary characteristics of bridge vibration. Thus, a novel wind-induced vibration response prediction model based on TCN for the high pier, large-span rigid bridge is proposed in this paper. TCN can effectively capture the long-distance dependence of bridge wind-vibration time series through the combination of causal convolution and dilated convolution. Causal convolution relies only on the current and past time steps in the prediction to avoid the leakage of future bridge vibration information. In contrast, dilated convolution overcomes the limitation of traditional convolutional networks in dealing with long sequences by expanding the receptive field so that the model can capture the dependency of longer horizons in fewer layers. TCNs have stable gradient propagation characteristics; the traditional RNNs and LSTMs are prone to suffer from gradient vanishing or exploding problems during training. TCN can effectively alleviate the problem of dealing with long sequence gradients through Residual Connections, which ensures the stable training of the model in the deep network. Moreover, TCN has the ability of parallel computing. RNN and LSTM rely on time-step recursive computation, which results in low training efficiency. TCN can process all input data in parallel within the same time step based on convolutional operation, which significantly improves computational efficiency.

## 2. Feature engineering

The dynamic monitoring data during the construction stage of a high-pier, large-span rigid bridge in Xinjiang, China, are employed to investigate the wind-vibration response prediction algorithm. The wind-vibration response prediction algorithm is investigated by using the dynamic monitoring data during the construction stage of a high-pier, large-span rigid bridge in Xinjiang, China. The bridge has a total length of 2292m, a deck width of 19m, a bridge height of 123.8m, and the main body is oriented east-west. The main girder span arrangement is 82 + 4 × 150 + 82 prestressed concrete continuous rigid structure, adopting longitudinal, transverse, and vertical three-way prestressed concrete structure. The hanging basket casting construction is divided into 18 construction sections to cast C60 concrete. The prestressing strand adopts a high-strength and low-relaxation strand in accordance with the national standard. The design adopts the strand with the nominal diameter of φs15.2mm and the standard value of tensile strength of fpk = 1860 Mpa. The lower pile foundation structure adopts variable section double-limb hollow pier casting C50 concrete, a maximum pier height of 114.8m. The bridge site has low mountain valley geomorphic features, the environmental category is Ⅱ, and the project structural engineering design reference period is 100 years. The bridge site has a temperate continental climate, with strong winds in spring and winter. According to the measured records, during the winter vacation in 2021, there were many times of windy weather for grade 11 at the construction site, which caused significant disturbance to the bridge construction, especially the main pier located at the entrance of the canyon. The characteristics of this bridge are highly representative of modern high-pier, large-span bridges. In this paper, the proposed model takes the mean wind speed, mean wind direction, and the mean squared error of the fluctuating wind speed as inputs, and by selecting the RMS values of the transverse bridge and along the bridge, vertical and torsional vibration as model ouputs and combining them with the actual monitoring data under the complex climate conditions in Xinjiang, the proposed model is verified to be widely applicable to similar structures.

The measured bridge vibration acquisition using JM3873G wireless vibration node, each accelerometer built-in two high-precision low-frequency pickup (vertical, horizontal optional) frequency of 64 Hz, the acceleration ranges of ± 5.767 *m*/*s*^2^, the resolution of the over-sampling mode, equipped with GPS own precise positioning and accurate timing, to achieve multiple wireless synchronization, the distance does not restrict the mode of communication for the remote wireless transmission. The collected data is uploaded to the supporting cloud platform to reflect the data collection situation and working status in real-time.

The wind environment data collection at the bridge site uses CJ-SWD3 three-dimensional ultrasonic wind speed and wind direction sensor and RS-CFSFX2 two-dimensional wind speed and wind direction sensor, the three-dimensional ultrasonic wind speed and wind-direction sensor collection frequency is 64 Hz, the wind speed range is 0 ~ 70 *m*/*s*, the accuracy is 2% at 12 *m*/*s*, the resolution is 0.01 m/s; the wind angle range is 0–359°, the wind angle range is 0–179°, the wind precision is 3° at 12 *m*/*s*, the wind angle range is 3° at 12 m/s, the wind accuracy is 3° (at 12 *m*/*s*), the wind accuracy is 3° at 12 *m*/*s*, the wind accuracy is 3° at 12 *m*/*s*. Wind direction accuracy is 3° at 12 *m*/*s*, and wind direction resolution is 1°. Two-dimensional ultrasonic wind speed and direction sensor acquisition frequency of 64 Hz, wind speed range of 0 ~ 70 *m*/*s*, accuracy of 0.2 *m*/*s*, resolution of 0.01 *m*/*s*; wind angle range of 0 ~ 359°, wind angle of attack range of 0–179°, wind accuracy of 3° at 12 *m*/*s*, wind resolution of 1°. The wind characteristic data is remotely transmitted to the supporting cloud platform, reflecting the data collection and working status in real-time.

**Fig 1:**
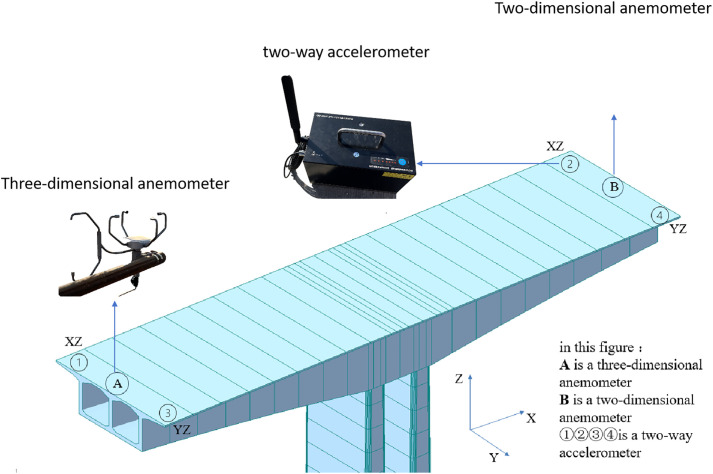
The schematic of the monitoring equipment layout of the bridge.

**Fig 2:**
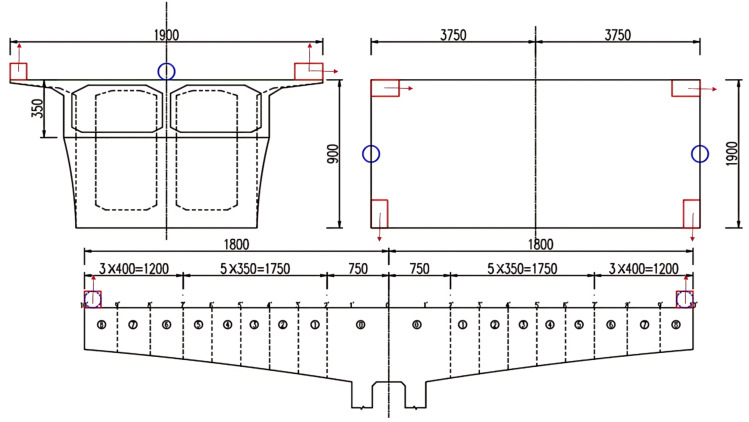
The dimension marking for the schematic diagram.

The monitoring equipment was installed at the end of the cantilever on both sides of the main pier to predict the vibration response of the bridge accurately. A total of four accelerometers, a three-dimensional anemometer, and a two-dimensional anemometer are arranged at the quarter position of the cantilever on both sides of the bridge. The specific instrument arrangement is illustrated in Fig 1, where each accelerometer has built-in vertical and horizontal 2-way high-precision low-frequency pickups. The dimension marking for the schematic diagram is shown in Fig 2. The horizontal direction of the two accelerometers on the same side points to the transverse-bridge and along the bridge directions, respectively. The vertical direction is perpendicular to the bridge deck, and the downstream and cross-bridge acceleration can be measured directly. The average value of the bridge vibration measured by two 2D accelerometers of the same cross-section in the vertical vibration acquisition direction is taken to measure the vertical bridge vibration response. Two 2D accelerometers of the same cross-section of the vertical vibration acquisition direction of the bridge vibration are measured by taking the difference and divided by the bridge width in order to measure the torsional direction of the bridge vibration response.

In summary, this monitoring system can obtain the four-way acceleration (torsion, vertical, transverse-bridge acceleration, and along the bridge acceleration) at the cantilever quarter-span position. The two anemometers measure the three-dimensional wind field at the bridge deck position. The three-dimensional anemometer is arranged at the west quarter position of the bridge abutment to obtain the instantaneous values of the three-dimensional wind speed, wind direction, and wind attack angle. The two-dimensional anemometer was arranged at the east quarter of the bridge abutment to obtain the instantaneous values of two-dimensional wind speed and wind direction angle.

### 2.1. Wind characteristic analysis

The wind dataset recorded from the eastern side of the cantilever was explicitly selected for detailed analysis. Wind speed measurements were acquired at a sampling frequency of 64 Hz.

#### 2.1.1. Average wind speed and wind direction.

**Fig 3:**
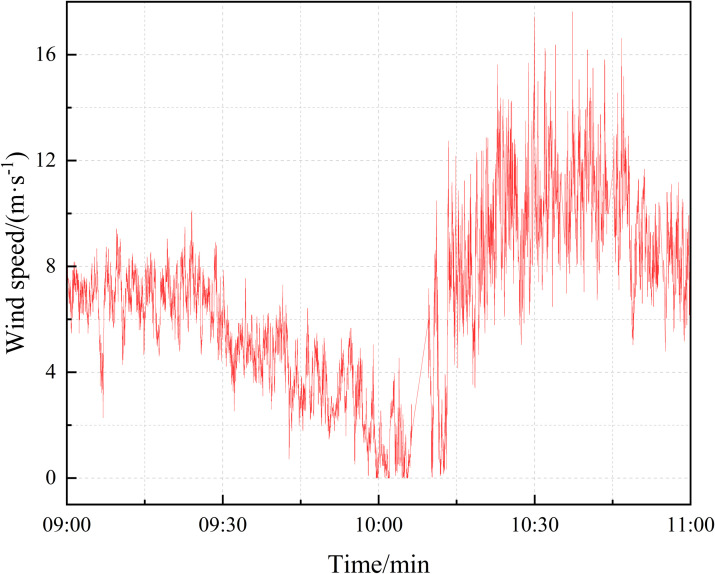
Measured mean wind speed time series curve.

**Fig 4:**
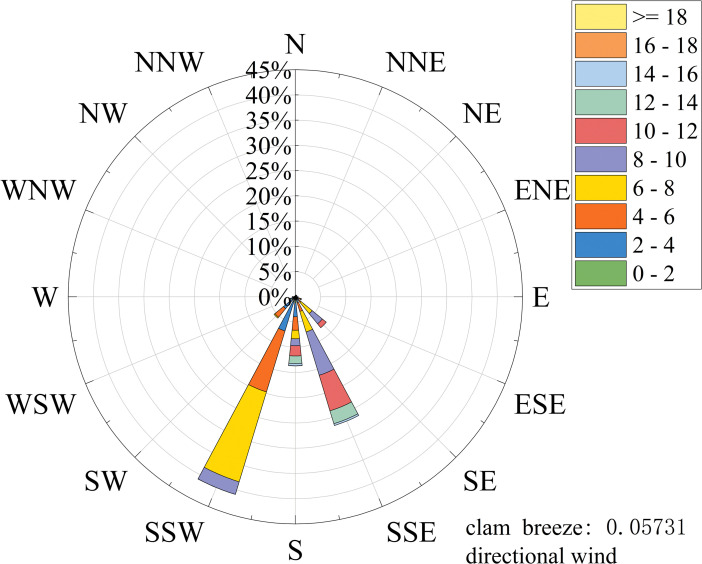
The rose chart of mean wind direction characteristics.

As depicted in Fig 3, the recorded wind speed at the quarter position of the main beam exhibited a gradual decrease between 9 a.m. and 10 a.m., followed by a rapid escalation beginning at 10 a.m. and reaching nearly 12 m/s within a brief period. The peak wind speed reached 17 m/s, with the gale persisting for approximately 40 minutes. The wind angle is defined as the angle between the projection of the prevailing wind direction on the horizontal plane and the northward direction. The zero-degree reference is the due north direction, and positive angles are assigned in the clockwise direction. An examination of Fig 4 reveals the prevalence of South South-West (SSW) and South South-East (SSE) as the dominant wind directions, comprising 40.9% and 26.3% of the total distribution, respectively. It indicates that the wind direction is concentrated.

#### 2.1.2. Turbulence intensity.

Turbulence intensity is straightforwardly related to the turbulent energy in the wind speed. It is one of the core parameters for predicting the wind vibration response, which quantifies the change in wind speed at a given time and is defined as follows:


$$I_{i}=\frac{\sigma_{i}}{U}(i=u,v)$$ (1)

where $I_{i}$ represents the intensity of the turbulence, $\sigma_{i}$ is the standard deviation of the pulsation component, and U denotes the average wind speed per unit time.

**Fig 5:**
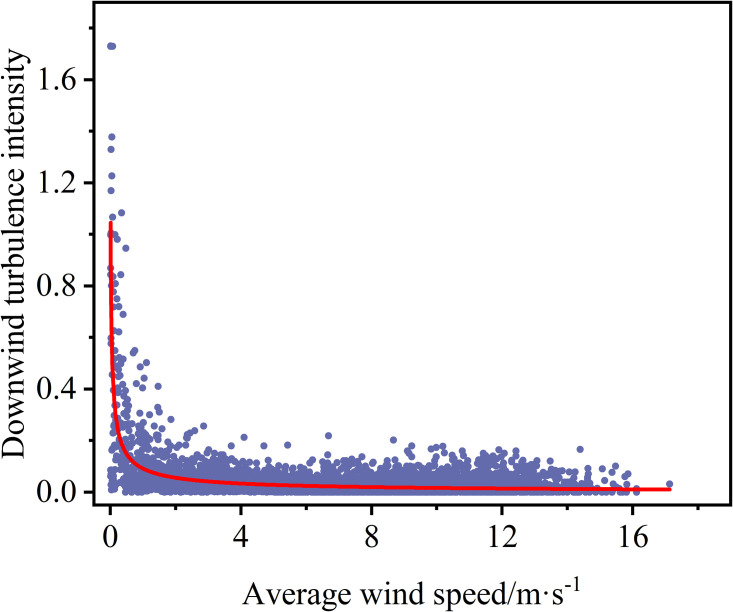
The scatter plot of the correlation between turbulence and mean wind speed for downwind turbulence intensity.

**Fig 6:**
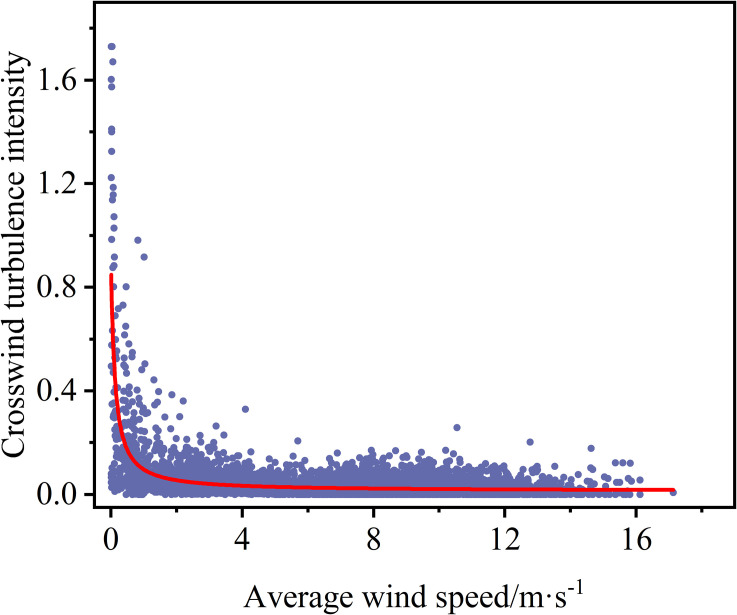
The scatter plot of the correlation between turbulence and mean wind speed turbulence intensity in the crosswind direction.

The selected measured wind longitudinal turbulence intensity ($I_{u}$) and transverse turbulence intensity ($I_{u}$) are plotted against the mean wind speed according to Eq. (1), as shown in Figs 5 and 6. We can learn that both longitudinal and lateral turbulence intensities show a declining trend with increasing average wind speed, and the degree of discretization gradually becomes smaller. The average values of $I_{u}$ and $I_{v}$ are 0.038 and 0.034, respectively. The ratio is that $I_{u}:I_{v}=1:0.895$, which is close to the recommended value of 0.88.

### 2.2. Vibration response analysis

The acquisition frequency of acceleration response is set to 64 Hz to ensure time resolution and accuracy of data acquisition. The mean wind speed, mean wind direction, and the mean squared error of fluctuating wind speed were selected as the input features of the machine learning dataset, while the RMS values of transverse bridge acceleration ($a_{H}$), along the bridge acceleration ($a_{L}$), vertical acceleration ($a_{V}$), and torsional acceleration ($a_{T}$) were used as the output features. The output transverse bridge acceleration ($a_{H}$) and along the bridge acceleration ($a_{L}$) can be obtained from the acceleration sensor.

The vertical acceleration ($a_{V}$) can be computed as:


$$a_{V}=\frac{V_{1}+V_{2}}{\text{2}}$$ (2)

Similarly, the torsional acceleration ($a_{T}$) can be written as:


$$a_{T}=\frac{V_{1}-V_{2}}{B}$$ (3)

where $V_{1}$ and $V_{2}$ denote the vertical acceleration on the left and right side of the main beam, respectively, in units of *m*/*s*^2^, and *B* is a constant whose value is the horizontal spacing in meters between two accelerometers of the same cross-section, which is taken to be 19 in this paper. Torsional acceleration is positive clockwise and negative counterclockwise.

**Fig 7:**
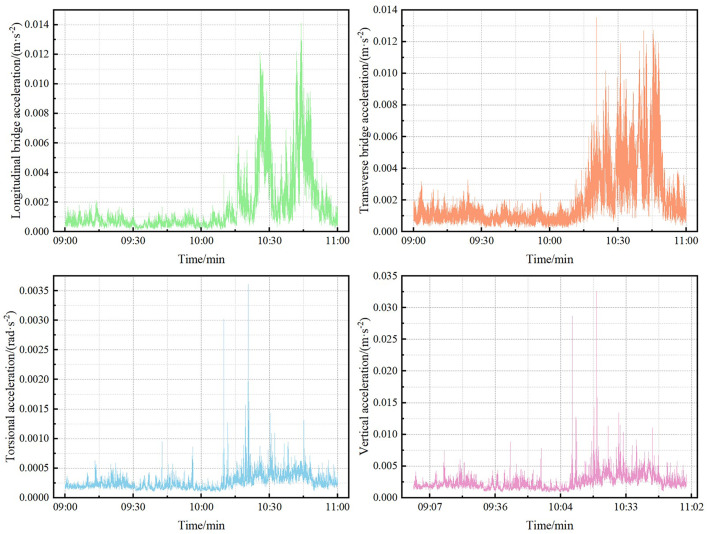
The RMS curve of measured vibration response acceleration in each direction.

The acceleration response curve shown in Fig 7 indicates that an increase in wind speed is accompanied by a significant increase in the acceleration response value in each direction, with the torsional acceleration having a relatively minor influence on the wind vibration response of the bridge.

### 2.3. Partial autocorrelation analysis

**Fig 8:**
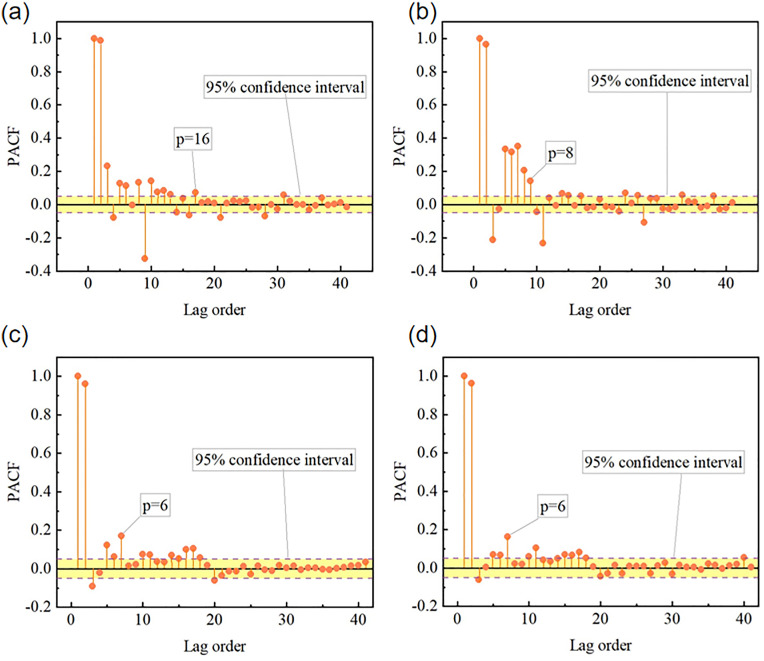
Distribution of partial autocorrelation functions for acceleration in different directions. (a) Lag order of acceleration in the along the bridge direction; (b) Hysteresis order of acceleration in the transverse direction; (c) Lag order of vertical acceleration; (d) Torsional acceleration hysteresis order.

The partial autocorrelation function (PACF) of the root mean square (RMS) values of acceleration is employed in this paper to investigate the effect of historical data on the current wind vibration response of bridges. Samples exceeding the 95% confidence interval are considered to exhibit significant correlation, while those within the confidence band are deemed insignificant. The lag order *p* represents the number of previous time steps influencing the current acceleration response. It is determined based on the truncated or trailing characteristics of the PACF. The values of PACF and *p* are shown in Fig 8. It can be seen from Fig 8a that the PACF plot exhibits significant partial autocorrelation at the first 16 lag steps. It suggests that the acceleration along the bridge direction has a strong memory effect, meaning the current acceleration is influenced by a longer sequence of historical data. Fig 8b shows that the lag order is identified as p = 8, which is relatively shorter than the along the bridge direction. It implies a moderate memory effect, where historical wind vibration responses still influence the current state, but the correlation decays more quickly. In Fig 8c, the PACF analysis determines a lag order of 6 steps. The lower value of p indicates that vertical accelerations are less influenced by long-term historical responses, which may be attributed to the dominant natural vibration modes and the energy dissipation characteristics of the structure in this direction. The PACF analysis determines a lag order of 6 steps in Fig 8d. This result suggests a similar autocorrelation behavior to the vertical direction, likely due to the coupling effects between torsional and vertical modes caused by wind excitation. A sliding window method (SWM) was adopted to construct the training samples. The length of the input sequence interval for each sliding window is *p*. The value of *p* is determined by the partial autocorrelation function of the isotropic acceleration RMS values.

## 3. The prediction model for wind vibration response based on TCN

The TCN model is primarily comprised of causal convolution, dilation convolution, and residual concatenation.

### 3.1. Causal convolution

Causal convolution is used to process time series data with a causal relationship between inputs and outputs. The forecasted value $y_{t}$ at a given moment *t* is only related to the da*t*a $x_{t}$ at moment *t* and the previous data $\left(x_{0},x_{1},\cdots ,x_{t-1}\right)$ before the moment *t*, i.e., the current s*t*atus is not influenced by the future information. At the same time, *t*he causal convolution is zero-padded at the beginning of the data sequence, which ensures that the lengths of the inputs and outputs are equal. Specifically, given an input sequence $X=\left(x_{0},x_{1},\cdots ,x_{t-1}\right)$, an output sequence $Y=\left(y_{0},y_{1},\cdots ,y_{t}\right)$, and a convolving kernel $H=\left(h_{0},h_{1},\cdots ,h_{k}\right)$, the result of the causal convolution for input $x_{t}$ can be expressed as:


$$y_{t}=(H*X{)}_{\left(x_{t}\right)}=\sum_{k=0}^{K}h_{k}\cdot x_{t-K+k}$$ (4)

where * denotes the causal convolving operation, $h_{k}$ is the weight of the convolving kernel, and *K* is the size of the convolving kernel.

Its receptive field can be expressed as [35]:


RF=1+(K−1)L (5)

where *RF* is the receptive field size, and *L* is the total number of network layers.

**Fig 9:**
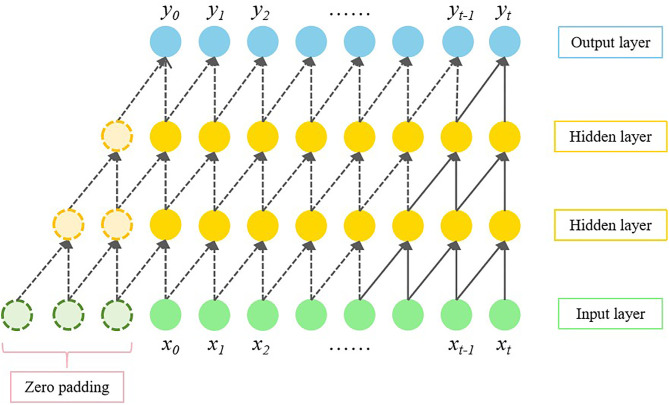
Schematic of one-dimensional causal convolution.

The schematic diagram of a one-dimensional causal convolution is shown in Fig 9.

### 3.2. Dilated convolution

To capture information on longer time scales, a dilated convolution operation based on causal convolution is applied. The input data to the convolution is expanded by interval sampling the sensory field, and it introduces an expansion rate into the convolution kernel. The convolution after expansion at $x_{t}$ can be expressed as:


$$y_{t}=(H*X{)}_{\left(x_{t}\right)}=\sum_{k=0}^{K}h_{k}\cdot x_{t-(K-k)d}$$ (6)

Where the expansion rate *d* typically scales exponentially with the depth of the network as:


$$d=2^{i}$$ (7)

where *i* represents the number of network layers.

The size of the expanded receptive field can be expressed as:


RF=1+(K−1)d (8)

**Fig 10:**
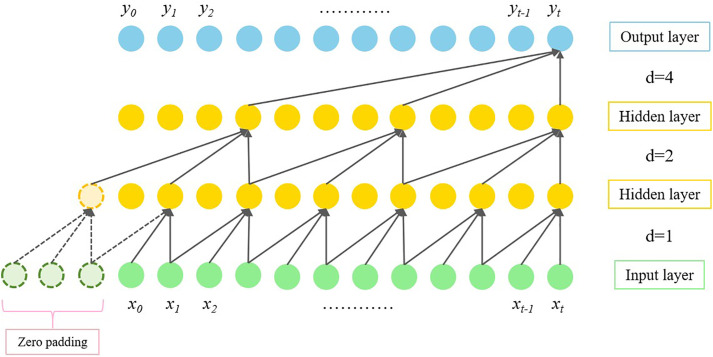
Principle schematic of dilated convolution.

The sensory field can be flexibly tuned by changing the size of the convolution kernel, the number of network layers, and the expansion rate. The main principle of dilated convolution is shown in Fig 10.

### 3.3. Residual concatenation

**Fig 11:**
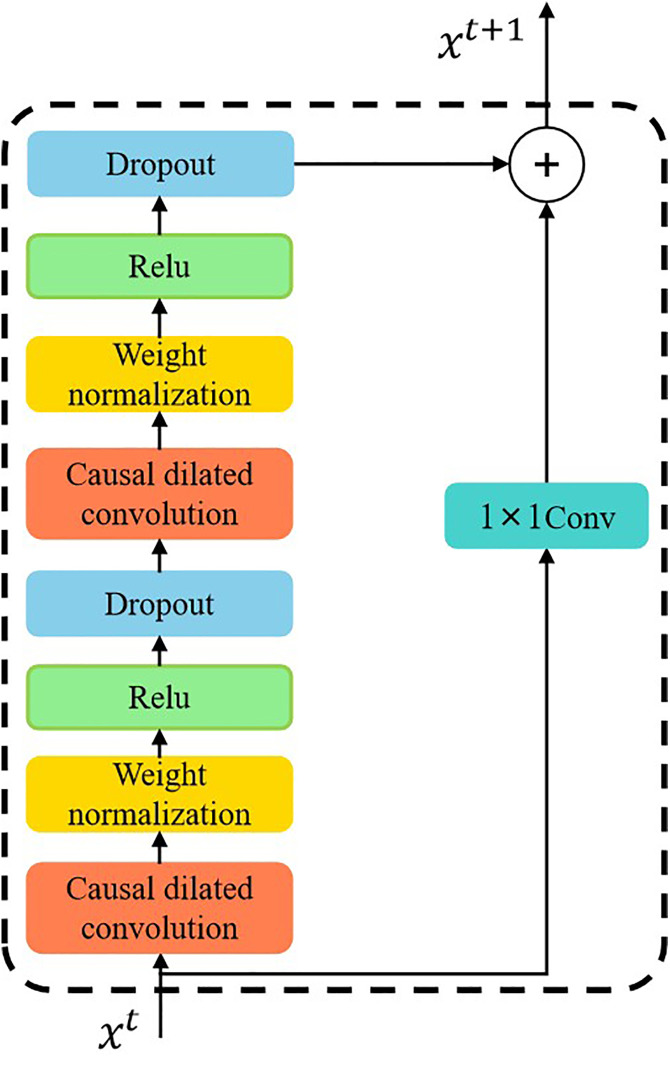
The structure of the residual connection block.


The TCN model increases the sensory field by incorporating more hidden layers, a deep network can negatively impact the stability of model training, leading to issues such as gradient vanishing and gradient explosion. Fortunately, the residual connections are incorporated into the TCN model with a view to increasing the depth of the network layers while also guaranteeing its accuracy through the use of residual blocks [36], whose specific structural configuration is depicted in Fig 11. It enables the model to learn the changes in the input sequence and facilitates the transfer of gradients, thereby mitigating problems such as gradient vanishing. The formula for this process is as follows:

y=x+F(x) (9)

Where F(x) is the output of the network layer, *x* is the input, and *y* is the final output.

The advantage of residual connectivity is that it allows the network to learn the residuals, i.e., the residual information that the model requires to learn, by directly adding connections across layers. It facilitates the transfer of information more efficiently in TCN models, which are designed to model deep networks and long sequences. Consequently, they capture long-term dependencies in sequences in real-world time series tasks more efficiently.

### 3.4. Model building

**Fig 12:**
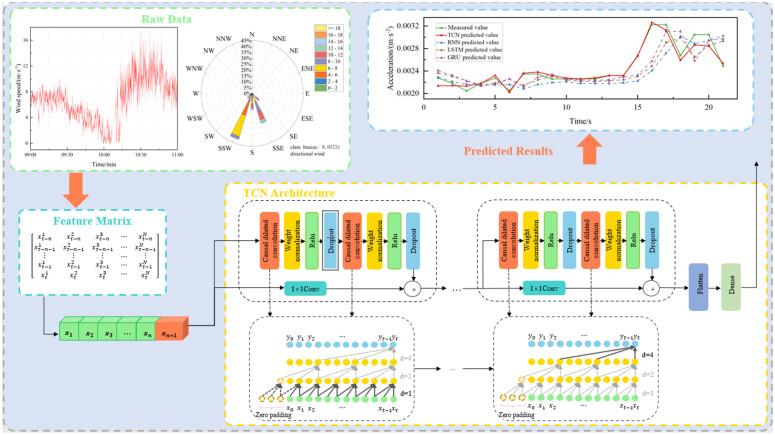
The framework diagram of the bridge wind-vibration response prediction algorithm.

The framework of the wind-vibration response prediction algorithm for bridges based on a TCN is proposed in this paper, and it is depicted in Fig 12. In the proposed deep learning model, the following three features are selected as input parameters to predict the response characteristics of the bridge under the action of the wind field: average wind speed, average wind direction, and mean squared error of pulsating wind speed. The objective of the algorithm is to predict the wind vibration acceleration response of bridges with greater efficacy by processing and analyzing wind speed and wind direction data. The algorithm begins by preprocessing the raw wind speed, wind direction, and wind vibration response data of the bridge. Median filtering is employed to remove spike noise, while outliers are identified using the interquartile range (IQR) rule and corrected via linear interpolation. Missing values are filled using linear interpolation to maintain temporal continuity. Crucial wind parameter features, such as mean wind speed, wind direction, wind angle of attack, and turbulence, are then extracted through statistical and correlation analyses. It then correlates the wind parameter features with the root-mean-square (RMS) of the wind acceleration response of the bridge. Correlation analysis was employed to identify the features that exhibited the strongest correlation with the RMS of wind vibration acceleration response of the bridge. These features were then selected to construct the feature matrix. The sliding window mechanism was utilized to establish the model training dataset. In the model training stage, the constructed feature matrix is employed as input to train the TCN model. It effectively reduces the number of parameters of the model. It improves the training efficiency and generalization ability of the model through the use of structures such as causal dilated convolution and residual linkage while capturing the long-time dependencies in the data. The TCN model consists of multiple residual blocks, each composed of two convolutional layers with increasing dilation rates. Specifically, the first residual block has 48 filters, a kernel size of 4, and a dilation rate of 1; the second residual block has 24 filters, a kernel size of 4, and a dilation rate of 2; and the third residual block has 16 filters, a kernel size of 4, and a dilation rate of 4. After each residual block, a dropout layer with a rate of 0.1 is applied to prevent overfitting. The model is then flattened, and a fully connected layer with a ReLU activation function is added afterward. During the training process, appropriate loss functions and optimization algorithms are employed to iteratively update the parameters of the model, thereby enabling the model to predict the wind vibration acceleration response of the bridge accurately.

## 4. Experiments and discussions

### 4.1. Data processing workflow

#### 4.1.1. Data cleaning.

**Fig 13:**
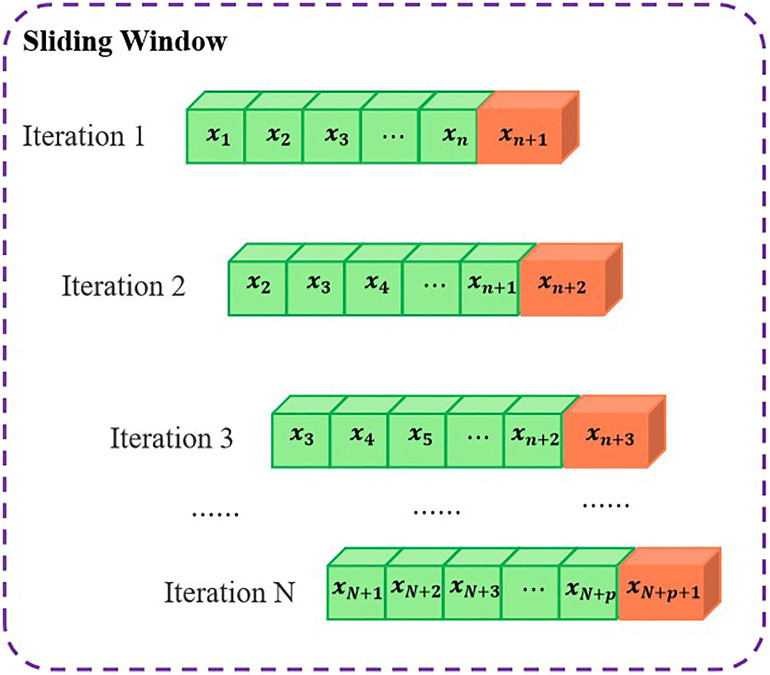
The illustration of sliding window for median filtering.

Multivariate wind speed prediction necessitates the consideration of a multiplicity of factors that affect wind speed. These factors exhibit distinctive physical properties and are quantified in distinct units of measurement. Direct analysis of unprocessed data may influence the accuracy of the outcomes. The raw monitoring data often contains noise, outliers, and missing values, primarily caused by sensor malfunctions or environmental interference. The median filtering was employed to effectively reduce the spike noise that may occur in the wind speed data. Median filtering is robust to extreme values and effectively removes isolated noise spikes by replacing each data point with the median value of its neighboring points within a sliding window. The sliding window is illustrated in Fig 13. The interquartile range (IQR) rule was utilized to identify outliers and apply linear interpolation to correct them. This approach ensures that the temporal continuity of the wind speed data is maintained without introducing abrupt deviations. Missing values in the raw monitoring data, caused by sensor failures or transmission errors, are filled using linear interpolation. The input features taken in this paper are obtained from the correlation coefficient matrix analysis.

#### 4.1.2. Feature selection.

In order to enhance the efficacy of the model in terms of training and prediction, the parameters exhibiting a low degree of correlation were removed through a correlation analysis of the measured wind field parameters and the wind vibration response of the bridge [37].

The correlation coefficients between the measured wind field parameters and the root mean square of the wind vibration response of the bridge are shown in Fig 11. The main correlation parameters of the wind vibration response of the bridge are determined based on the correlation magnitude of the parameters so as to minimize the redundant input variables for the subsequent neural network modeling. Where *U* denotes the average wind speed; α represents the mean wind angle; $\sigma_{u}$ and $\sigma_{v}$ are the mean square deviation of pulsating wind speed in the downwind direction and crosswind direction, respectively; $I_{u}$ and $I_{v}$ represents the turbulence intensity in the downwind direction and crosswind direction, respectively; and $a_{H}$, $a_{L}$, $a_{V}$, $a_{T}$ indicates the RMS values of the transverse, along the bridge, vertical, and torsional accelerations for the main beam, respectively.

**Fig 14:**
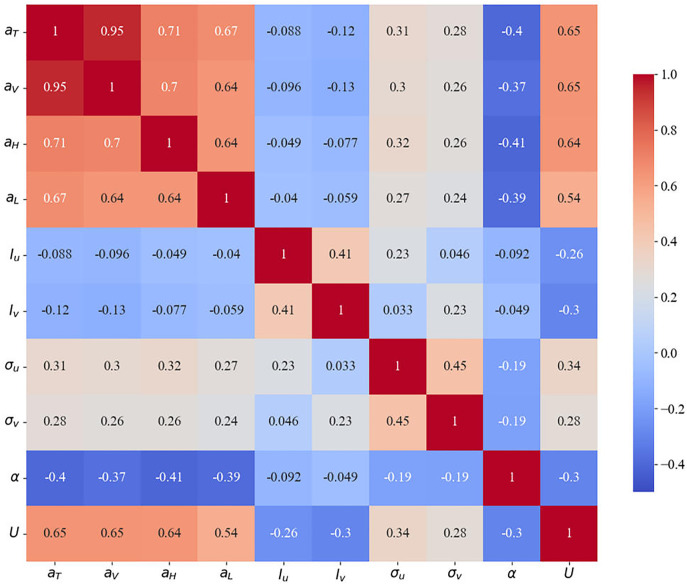
Correlation coefficients between wind characteristic parameters and RMS values of wind vibration response of bridges.

As illustrated in Fig 14, the strongest correlation is observed between the mean wind speed and the bridge wind vibration response, with a correlation coefficient of approximately 0.65. The mean wind direction exhibits a higher correlation with the bridge wind vibration response, with a correlation coefficient of approximately 0.4. The correlation coefficient between the mean squared error of the pulsating wind speed and the bridge wind vibration response is approximately 0.3. However, the correlation coefficient between turbulence intensity and wind vibration response is relatively low, indicating a limited contribution to bridge wind vibration. Therefore, the data set of the prediction model was constructed using the mean wind speed, mean wind direction, and mean squared error of pulsating wind speed as input parameters, and the main girder transverse direction, transverse direction, vertical direction, and torsional acceleration as output parameters.

#### 4.1.3. Training sample construction.

The dataset used in this study was collected from time-series wind-induced vibration response data from a bridge structural health monitoring system. The data were divided into training, validation, and test sets in the ratio of 80%, 10%, and 10%. The training set was used to optimize the model parameters, the validation set was used for hyper-parameter tuning, and the test set was used to evaluate the predictive performance of the model on unseen data. The dataset division was kept consistent with the wind speed and direction for the downbridge acceleration, transverse acceleration, vertical acceleration, and torsional acceleration.

#### 4.1.4. Normalization.

It is imperative to normalize the data, which entails adjusting different variables to comparable magnitudes to guarantee the accuracy and stability of the model [38]. The normalization process facilitates the simplification of the model’s processing flow on the data, thereby enhancing the model’s processing efficiency and accelerating convergence. In this section, the min-max normalization technique is employed, which converts the initial data into the range of 0–1 through a linear transformation. The calculation of normalization is illustrated as follows:


$$x^{\prime }=\frac{x-x_{\min}}{x_{\max}-x_{\min}}$$ (10)

Where *x* is the original data, *x*’ denotes the normalized data, $x_{\min}$ is the minimum value of variable *x*, and $x_{\max}$ is the maximum value of variable *x*.

#### 4.1.5. Model training.

For the purpose of verifying the prediction accuracy and generalization of the proposed Temporal Convolutional Network (TCN) model, a comprehensive experimental comparison was conducted with several state-of-the-art algorithms, including Recurrent Neural Networks (RNN), Long Short-Term Memory (LSTM), and Gated Recurrent Unit (GRU) models. The training process was conducted with the same training dataset and executed in the same hardware environment to ensure fairness in the experiments. To ensure fairness in experimental comparisons, all four proposed TCN models employed identical parameter configurations for predicting acceleration in various directions, and the Adam algorithm was employed to optimise the model training process. Hyperparameters for benchmark algorithms, such as RNN, LSTM, and GRU, were also optimised using a grid search method. The training was carried out over multiple iterations with early stopping to prevent overfitting, and the model’s performance was monitored in real time using a validation set. The training epochs, optimizer learning rate, and other hyperparameters were uniformly configured across all TCN models.

#### 4.1.6. Evaluation indicators.

Finally, the prediction accuracy of each model on the test set is evaluated using performance metrics such as Mean Absolute Error (MAE), Mean Absolute Percentage Error (MAPE), Root Mean Square Error (RMSE), and Coefficient of Determination (R^2^) Dynamic Time Warping (DTW) in order to comprehensively compare their performance in predicting bridge acceleration and to validate their prediction accuracy and generalization ability. The formula for each machine learning traditional evaluation metric is as follows:


$$MAE=\frac{\text{1}}{N}\sum_{t=1}^{N}|y_{t}-{\hat{y}}_{t}|$$ (11)

$$RMSE=\sqrt{\frac{\text{1}}{N}\sum_{t=1}^{N}{\left(y_{t}-{\hat{y}}_{t}\right)}^{2}}$$ (12)

$$MAPE=\frac{\text{1}}{N}\sum_{t=1}^{N}\left|\frac{y_{t}-{\hat{y}}_{t}}{y_{t}}\right|\times 100\%$$ (13)

$$R^{2}=1-\frac{\sum_{t=1}^{N}{\left(y_{i}-{\hat{y}}_{i}\right)}^{2}}{\sum_{t=1}^{N}{\left(y_{i}-\bar{y}\right)}^{2}}$$ (14)

Where $y_{t}$ denotes the measured value of acceleration at moment t; ${\hat{y}}_{t}$ denotes the predicted value of acceleration at moment *t*; *y* denotes the mean acceleration value; *N* is the total number of samples.

DTW is a nonlinear sequence alignment method that computes the shortest cumulative distance between the predicted and actual series while allowing for local time stretching or compression. The core idea is as follows:

1) Cumulative distance matrix construction

Given an actual time series $y=\left\{y_{1},y_{2},...,y_{n}\right\}$ and the prediction sequence $\hat{y}=\left\{{\hat{y}}_{1},{\hat{y}}_{2},...,{\hat{y}}_{m}\right\}$, a n×m local distance matrix was constructed first:


$$D(i,j)=(y_{i}-{\hat{y}}_{j}{)}^{2}$$ (15)

where D(i,j) denotes the actual sequence *ith* point of the actual sequence and the predicted sequence *jth* point of the predicted sequence.

2) Shortest Path Calculation

Based on the local distance matrix D(i,j), the cumulative distance matrix is calculated by dynamic programming C(i,j):


C(i,j)=D(i,j)+min{C(i−1,j),C(i,j−1),C(i−1,j−1)} (16)

Where C(i,j) denotes the minimum cumulative distance from the starting point (1,1) to the endpoint (i,j).

3) Normalized distance of the shortest path

The DTW distance is defined as the normalized value of the cumulative distance on the path from the start point to the endpoint:


$$DTW=\frac{C(n,m)}{n+m}$$ (17)

Normalization removes the effect of sequence length differences and makes the DTW distances more comparable.

### 4.2. Hyperparameter optimization

The hyperparameters have a significant effect on the performance of the proposed wind vibration response prediction algorithm based on TCN. In this paper, the grid search method is employed to optimize the hyperparameters of the model. Each hyperparameter is set with a reasonable range of values and different combinations of hyperparameters are selected through multiple exhaustive trials to train the model. The hyperparameter configuration corresponding to the trial with the best predictive performance in the validation set is considered as the optimal solution. In addition, adjusting the number of ephemeral elements in model training is a key aspect of neural network modeling. too many epochs may lead to overfitting of the model, while too few epochs may prevent the model from learning sufficiently and lead to underfitting. Both overfitting and underfitting can hinder the model’s predictive performance on test data. To address this issue, we introduce an early stopping strategy by setting a 20-calendar-element early stopping criterion: if the model’s performance on the validation set does not improve within 20 consecutive calendar elements, training is stopped and the model with the highest validation accuracy within that calendar element is considered as the final result. The experimental results show that the optimized TCN model can effectively capture the complex dynamic characteristics of bridge wind vibration response and significantly improve prediction accuracy. Table 1 shows the range of hyperparameters and their optimal values.

**Table 1:** The range of hyperparameters and results.

Hyperparameter	Value Range	Optimal Value
Batch Size	16–128	96
Filters in Output	16–128	64
Layers Number	3–7	4
Dropout	0.1 to 0.5	0.2
Learning Rate	0.1,0.01,0.001,0.0001	0.01
Kernel Size	2–8	5

### 4.3. Comparison of predictive effects of different models

**Fig 15:**
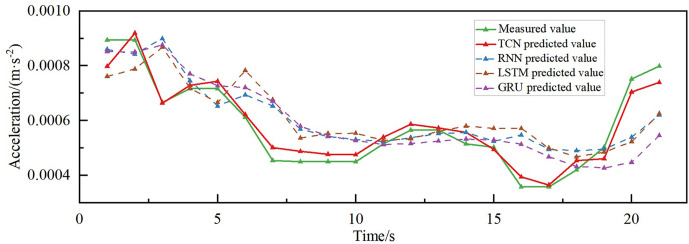
Acceleration prediction results of different models along the bridge acceleration.

**Fig 16:**
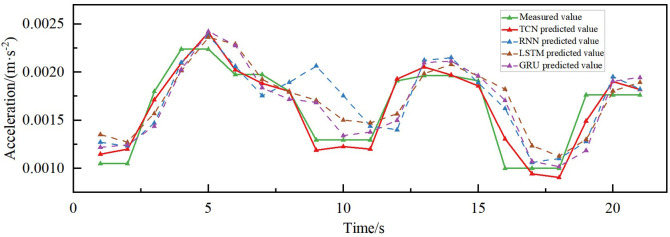
Acceleration prediction results of different models in transverse acceleration.

**Fig 17:**
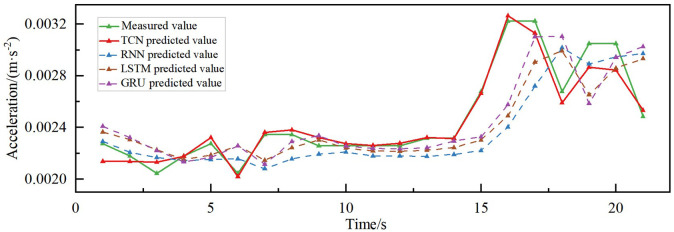
Acceleration prediction results of different models in vertical acceleration.

**Fig 18:**
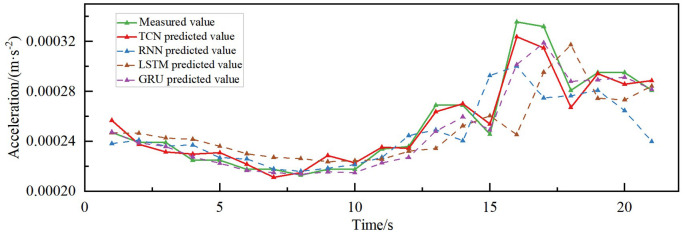
Acceleration prediction results of different models in torsional acceleration.

The combined acceleration prediction results of different models in different directions are shown in Figs 15–18. According to the comparison of the matching effect of the four models, the prediction effect of the TCN model is better than the other compared models, the prediction effect of the RNN model is the worst, and the prediction results of the LSTM and GRU models are in the middle between the TCN and RNN models. To better compare the prediction performance of different models, the error indices of different models are calculated separately, as shown in Table 2.

**Table 2:** Comparison of prediction errors of different.

Accelerations	Predictive model	MAE/10^–5^	R^2^/%	RMSE/10^–5^	MAPE/%	DTW Distance
Along the bridge	TCN	9.98 *	88.62*	13.46 *	13.6 *	0.0061*
	RNN	12.39	82.67	16.49	16.0	0.0112
	LSTM	10.36	88.38	14.51	14.8	0.0113
	GRU	10.35	83.94	15.12	14.3	0.0103
Transverse	TCN	16.78 *	71.34*	23.13 *	13.6 *	0.0129*
	RNN	20.35	65.40	27.73	15.9	0.0159
	LSTM	18.13	68.05	25.36	14.5	0.0162
	GRU	17.30	67.43	24.49	14.0	0.0182
Vertical	TCN	11.54 *	70.18	17.79 *	4.9 *	0.0060*
	RNN	14.66	70.81	21.10	6.2	0.0070
	LSTM	12.73	71.93	18.77	5.4	0.0060
	GRU	13.14	73.14	18.53	5.2	0.0062
Torsional	TCN	1.58	68.00	2.00 *	4.9 *	0.00066*
	RNN	1.49	66.18	2.23	6.1	0.00093
	LSTM	1.45	68.00	2.14	5.8	0.00075
	GRU	1.46	71.18	2.16	5.6	0.00075

The MAPE of the prediction model based on TCN is less than 5% for both torsional acceleration and vertical acceleration, while the R^2^ reaches 70.18% and 88.62% in the vertical and along the bridge directions, respectively, indicating its high explanatory power, as can be seen in Table 2. Additionally, the MAE and RMSE errors of the proposed prediction model based on TCN are also superior to those of the RNN, LSTM, and GRU comparative models. The MAE, RMSE, and MAPE of the proposed TCN-based model in predicting the transverse bridgeward acceleration are 16.78, 23.13 and 13.6%, which are improved by 3.00%, 5.55%, and 2.86%, respectively, compared to the GRU model, and 17.54%, 16.59% and 14.47%, respectively, compared to the RNN model. In the prediction of along-the-bridge acceleration, the MAE, RMSE, and MAPE of the TCN model are 9.98, 13.46, and 13.6%, respectively, R^2^ up to 88.62%, which are the most effective of the four models. From the point of view of DTW distance analysis, the TCN model demonstrated optimal performance in predicting both along-bridge (0.0061) and vertical (0.0060) vibrations, which indicates that its predicted curves most closely resemble the actual curves in shape and exhibit the best temporal consistency. For transverse vibrations, although TCN still yields the smallest DTW value (0.0129), the differences among models are not significant, indicating that prediction in this direction is generally more challenging. For torsional vibrations, all models exhibited very low DTW values (0.00066–0.00093), indicating that waveforms to predict are highly consistent. TCN performed similarly to LSTM and GRU, slightly outperforming RNN. Overall, TCN demonstrates superior dynamic performance across most directions, particularly in along-bridge and vertical vibrations, highlighting its strong ability to capture temporal patterns.

The superior performance of the TCN model can be attributed to its unique structural characteristics. First, TCN employs causal convolution, which ensures that the model’s predictions only depend on past inputs, aligning with the physical nature of wind-induced vibration responses, where past wind conditions influence current vibrations. Second, the use of dilation convolution in TCN allows for a larger receptive field without increasing the number of parameters, which enhances the model’s ability to capture long-range dependencies in time series data, a critical aspect when predicting wind-induced vibrations that may involve long temporal lags. This capability is critical in scenarios where wind speed changes drastically over short periods, a condition that the TCN model can handle effectively due to its flexibility in adjusting the receptive field.

Moreover, TCN incorporates residual connections, which help mitigate the issue of vanishing gradients, ensuring stable training even for deeper networks. The results in better memory utilization and make TCN particularly well-suited for sequences with long-term dependencies. In contrast, RNN often encounters the problem of gradient vanishing or gradient exploding when dealing with long sequences, which leads to difficulties in capturing long-distance dependencies. LSTM alleviates the problem by introducing a gating mechanism to some extent, enhancing its capability to capture long-term dependencies in vibration responses. GRU has structurally refined the LSTM model to improve processing efficiency. TCN combines causal convolution, dilation convolution, and residual connection to achieve flexible adjustment of receptive fields, a high degree of parallelism, and stability of gradients, as well as a significant advantage in memory utilization.

After considering the prediction errors of the four models, it was concluded that the TCN model, with its unique structure, demonstrated an excellent prediction effect in wind vibration prediction in different directions of the bridge and could realize the prediction of wind vibration response in the scenario of large changes in wind speed in a short period. The prediction trend and the measured accuracy of the GRU and the LSTM models were roughly the same. However, the prediction effect was inferior to that of the TCN model and better than that of the RNN model.

**Fig 19:**
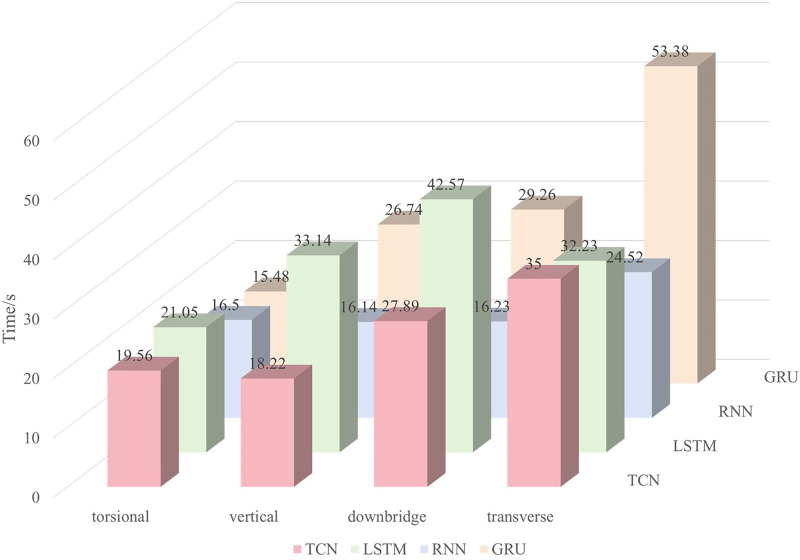
Comparison of training time.

Additionally, we recorded the training time, prediction time, and inference speed for each model, as shown in Tables 3–5 and Fig 19. The results indicate that RNN has the shortest training time, followed by TCN, while GRU takes the longest. However, despite its fast training time, RNN performs poorly in terms of evaluation metrics. In contrast, TCN strikes a good balance, offering moderate training time while achieving strong performance. The prediction times across the models are relatively similar. As for inference speed, TCN exhibits a slightly slower rate of 3ms per epoch. It mainly stems from the deep structure and dilated convolution operations that TCNs rely on to capture long-range dependencies, where the expansion of the receptive field comes at the cost of significantly increasing model depth and computational complexity. However, this inference speed can meet the requirements of real-time prediction of wind-induced vibration responses of bridges.

**Table 3:** Comparison of training time for different algorithms (Unit: second).

Time/s	TCN	LSTM	RNN	GRU
Along the bridge	19.56	21.05	16.50	15.48
Transverse	18.22	33.14	16.14	26.74
Vertical	27.89	42.57	16.23	29.26
torsional	35.00	32.23	24.52	53.38

**Table 4:** Comparison of forecast time for different algorithms (Unit: second).

Time/s	TCN	LSTM	RNN	GRU
Own-bridge	0.19	0.17	0.14	0.23
Transverse	0.19	0.17	0.15	0.19
Vertical	0.18	0.17	0.18	0.19
Torsional	0.19	0.16	0.15	0.19

**Table 5:** Comparison of inference speed for different algorithms (Unit: ms/patch).

Algorithm	TCN	LSTM	RNN	GRU
Inference speed	3	1	0.8	2

To further validate the statistical performance of the proposed algorithm, we conducted a paired t-test with a, which is shown in Table 6. As indicated in Table 6, the p-values for the paired t-tests of the vibration prediction results in the four different directions were all less than 0.05. And it is means that there were significant differences in the performance of the two algorithms, the TCN algorithm significantly outperforms the three benchmark algorithms at a Alpha level of α = 0.05.

**Table 6:** The p-value result of paired t-tests for different prediction algorithms(Alpha level of α = 0.05).

Compare algorithm	Along the bridge	Transverse	Vertical	Torsional
RNN	0.048828	0.007752	0.000027	0.001475
LSTM	0.000623	0.000278	0.000008	0.001482
GRU	0.029547	0.000155	0.000047	0.001429

### 4.4, Sensitivity experiment

**Fig 20:**
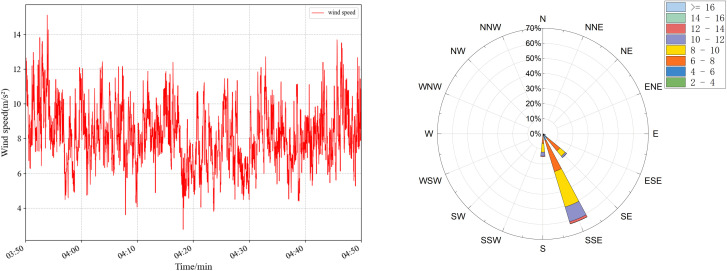
Wind speed (left) and wind rose plots (right) were used for sensitivity analysis of raw data for group a.

**Fig 21:**
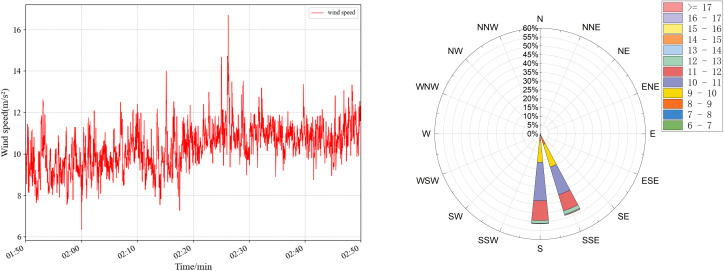
Wind speed (left) and wind rose plots (right) were used for sensitivity analysis of raw data for group b.

**Fig 22:**
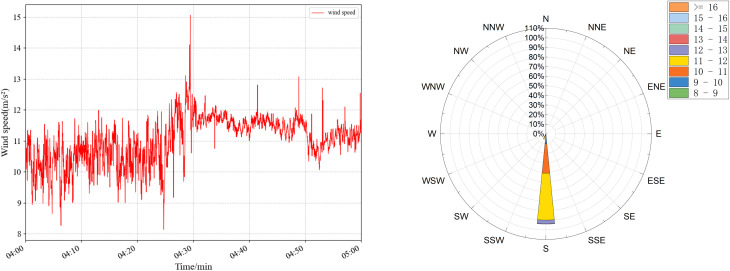
Wind speed (left) and wind rose plots (right) were used for sensitivity analysis of raw data for group c.

**Fig 23:**
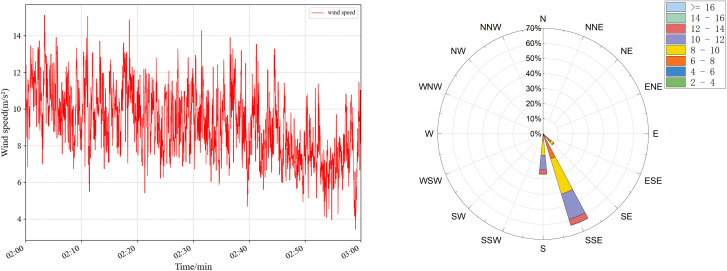
Wind speed (left) and wind rose plots (right) were used for sensitivity analysis of raw data for group d.

The wind-induced vibration response prediction faces great challenges in practical bridge engineering, especially in areas with complex and variable wind conditions, such as high mountain valleys. In order to evaluate the accuracy and sensitivity of TCN under different wind conditions, targeted experiments were designed and carried out. Four sets of wind speed data at different periods were selected, and the specific statistical characteristics are shown in Table 7 and Figs 20–23, which have significant differences and can effectively simulate diverse wind field environments. The performance of the TCN model under different wind speeds and fluctuation characteristics is systematically analyzed by comparing the prediction results of the along-the-bridge, transverse, torsional, and vertical acceleration responses with each data set and the sensitivity of the model to the changes of the input data and its robustness is further explored. The experimental results provide a theoretical basis and technical support for the prediction of wind vibration response of bridges under complex wind field conditions.

**Table 7:** Statistical characteristics of the four sets of wind data.

Group ID	Average wind speed	wind speed variance	Average wind rating	Characteristics of wind conditions
a	8.219	2.999	5	Lower wind speeds, significant fluctuations
b	9.209	3.431	5	Low wind speeds, sharp fluctuations
c	10.341	1.095	5	High wind speeds, low fluctuations
d	11.013	0.538	6	Higher wind speeds, slight fluctuations

The experimental results are shown in Table 8, and the predicted wind vibration response of the bridge is significantly correlated with the wind field characteristics. Under the low wind speed conditions for groups a and b, despite the significant wind speed fluctuations, the prediction results perform better in the vertical and torsional directions. The MAE in the torsional direction is only 1.23 × 10-⁵ for group a, and the MAE in the vertical direction for group b is 6.88 × 10-⁵. However, the errors in the lateral and transverse bridge directions are significant. The MAPE in the transverse direction of group b is as high as 19.37%. The performance of prediction errors tends to be more complex under high wind speed conditions for groups c and d, despite more minor wind speed fluctuations: group c performs optimally in the vertical direction with an MAE of 3.76 × 10-⁵. It has significantly higher errors in the cross-bridge direction with RMSE of 50.02 × 10- ⁵. In contrast, the prediction errors of group d remain low in most directions. Comprehensive analyses show that the magnitude of wind speed fluctuations and the overall wind speed level have an important influence on the prediction results in different directions. The overall stability of the model is high under high wind speed and low fluctuation conditions. However, the prediction ability of the model for complex directions, such as the cross-bridge direction, needs to be further improved under low wind speed and high fluctuation conditions.

**Table 8:** Sensitivity test results.

Group ID	Accelerations	MAE/10^–5^	RMSE/10^–5^	MAPE/%
a	vertical	11.89	15.07	6.75
	torsional	1.23	1.65	4.73
	along the bridge	19.13	24.04	12.55
	transverse	27.26	36.38	11.40
b	vertical	6.88	9.22	4.99
	torsional	0.98	1.19	6.47
	along the bridge	17.09	22.73	15.58
	transverse	23.35	28.56	19.37
c	vertical	3.76	4.83	9.50
	torsional	23.46	29.61	8.44
	along the bridge	23.98	31.46	8.68
	transverse	41.25	50.02	9.74
d	vertical	17.72	22.50	5.27
	torsional	1.81	2.25	5.00
	along the bridge	27.42	35.14	8.68
	transverse	12.81	16.01	10.51

The development phase of the prediction model requires supervised learning from historical wind and acceleration monitoring data, but once the model is trained and verified to be reliable, the prediction results can be output in subsequent engineering applications by simply inputting the wind field parameters, which can be obtained from weather forecast information without real-time acceleration monitoring. In addition, another application scenario of this prediction model is to provide a means of vibration prediction based on wind field parameters in scenarios where sensors cannot be deployed for a long period of time due to harsh environments, cost constraints, or where monitoring data is not yet available for new bridge structures.

## 5. Conclusions

Accurately predicting wind vibration response has always been an essential challenge in the field of bridge engineering, which can effectively guide engineering design and improve the safety and stability of barrier bridge structures. In this paper, a bridge wind-vibration response prediction model based on the temporal convolutional network is proposed, which integrates the parallel computing of CNN and the temporal modeling capability of RNN and which can more accurately exploit the nonlinear abstract characteristics of the bridge wind vibration data, and detailed comparative experiments are conducted to validate the model. The obtained conclusions mainly include:

(1) A specific correlation exists between the bridge wind vibration response and the wind speed, wind direction, and the mean square error of pulsating wind speed in areas with significant wind speed variations.
(2) The bridge wind-vibration response prediction algorithm based on TCN has high accuracy and stability, outperforming traditional models such as RNN, LSTM, and GRU in the prediction of transverse, longitudinal, vertical, and torsional directions. It can be adapted to wind vibration response prediction in scenarios with significant wind speed variations.
(3) The proposed method relies solely on basic wind field parameters and structural acceleration response data, maintaining low computational costs even under conditions with limited monitoring data. It provides an effective methodological foundation for the development of real-time early warning and intelligent monitoring systems for bridge wind-induced responses, with strong prospects for engineering applications.

In future research, the data set can be expanded by combining the field monitoring data and simulation technology to more accurately excavate the wind vibration mechanism of bridges under the scenario of significant changes in wind speed within a short period so as to provide theoretical support and technical guarantee for the safety management and risk control of the bridge project.

## Supporting information

S1 Data The dataset.
(RAR)
